# Critical Diagnostic Delay Thresholds in Breast Cancer: A Molecular Subtype-Based Causal Analysis From Saudi Arabia

**DOI:** 10.7759/cureus.81106

**Published:** 2025-03-24

**Authors:** Reem A Alsalamah

**Affiliations:** 1 Surgery, Qassim University, Qassim, SAU

**Keywords:** breast cancer, causal inference analysis, molecular oncology, saudi arabia, tumor biology

## Abstract

Introduction

Diagnostic delays in breast cancer can significantly affect treatment outcomes. Currently, the causal mechanisms and critical time thresholds remain poorly defined across the different molecular subtypes of breast cancer. We investigated the relationship between diagnostic delays and breast cancer outcomes based on the data from our center, with a focus on identifying actionable intervention points within the diagnostic pathway.

Methods

We conducted a retrospective cohort study of 802 breast cancer patients treated at King Fahad Specialist Hospital in Qassim Province, Saudi Arabia (2017-2024). Using directed acyclic graphs and mediation analysis, we quantified the causal pathways through which delays impact the outcomes. Markov chain modeling was utilized to determine the molecular subtype-specific critical thresholds where stage migration probability exceeds 10%.

Results

We found that 589 patients (73.5%) experienced high-risk delays (over two months). Stage migration emerged as the primary mediator, accounting for 67.3% (95% CI: 58.4-76.1%) of the total effect of delays on survival. We have identified multiple critical thresholds across molecular subtypes: 38 days for triple-negative, 52 days for HER2-positive, and 85 days for ER+/PR+/HER2- tumors. Hazard ratios for mortality increased progressively with delay duration, from 1.18 (95% CI: 1.05-1.32) for delays of two weeks to one month to 2.35 (95% CI: 2.06-2.67) for delays that are equal to or more than one year, translating to an average 3.40 life years lost per patient.

Conclusions

The impact of diagnostic delays on breast cancer outcomes is fundamentally governed by tumor biology, with significant vulnerability thresholds aligned with molecular aggressiveness. Our findings support applying a biologically informed triage system where molecular characteristics determine maximum acceptable diagnostic intervals. Using the suggested approach, we may achieve a better balance in the resource constraints with biological imperatives, and possibly improve survival outcomes without proportional increases in healthcare expenditure.

## Introduction

While diagnostic delays in breast cancer are widely recognized as detrimental to outcomes, the heterogeneity of their impact across different molecular subtypes remains poorly understood [1]. Recent evidence suggests that the biological aggressiveness associated with specific molecular profiles (triple-negative, HER2-positive, and hormone receptor-positive tumors) may create differential vulnerability to time delays between symptom onset and treatment initiation [2,3]. This biological variability potentially undermines the current one-size-fits-all approach to diagnostic pathways and urgency classifications [4]. Understanding the subtype-specific temporal thresholds beyond which stage migration significantly accelerates could transform triage protocols and resource allocation in breast cancer care, particularly in regions with increasing incidence rates and limited healthcare resources such as Saudi Arabia [5,6]. This knowledge gap represents a critical barrier to developing evidence-based, biologically informed diagnostic pathways that appropriately prioritize patients at the highest risk of delay-related progression. In this study, we aim to investigate the role of diagnostic delays, disease progression, and patient outcomes within the context of breast cancer care based on data collected from our research center.

The rising incidence of breast cancer in Saudi Arabia presents a significant public health challenge [2-4]. Over the past two decades, breast cancer rates have increased nearly tenfold, now accounting for approximately 29% of all new cancer cases among Saudi women [2]. This epidemiological shift has created an urgent need for optimized diagnostic and treatment pathways. Unlike Western countries, where organized screening programs have facilitated earlier disease detection [5], many patients in Saudi Arabia present with symptomatic disease, often at advanced stages [6,7]. Understanding the critical time-dependent nature of breast cancer progression and identifying actionable intervention points within this manner are essential steps toward improving outcomes in this population [8].

The relationship between diagnostic delays and breast cancer outcomes is multifaceted and impacted by various clinical and biological factors. Previous studies have documented the adverse impact of delays on stage at diagnosis, with consequent implications for treatment intensity and survival [9,10]. However, significant knowledge gaps persist regarding the underlying mechanisms and the heterogeneity of delay-related risks across different patient subgroups [9,10]. Of particular importance is the differential impact of delays across molecular subtypes, which remain incompletely investigated and poorly studied, especially in Middle Eastern populations [11,12]. The traditional statistical approaches have often failed to disentangle direct and indirect effects, limiting our understanding of how delays affect outcomes through various mediating pathways.

Within the Saudi healthcare system, multiple factors may contribute to diagnostic delays, including patient-related factors (symptom recognition, healthcare-seeking behavior), primary care factors (initial assessment, referral practices), and system-level factors (appointment wait times, diagnostic capacity, care coordination) [6,7]. The relative contributions of these factors and their interactions have not been evaluated in previous studies. Furthermore, the critical time thresholds at which delays significantly impact disease progression and survival outcomes remain poorly defined, especially in the manner of molecular subtype heterogeneity [6,7]. These limitations hinder the development of evidence-based triage protocols and targeted intervention strategies.

Our study looks to address these gaps through a directed and advanced analysis of breast cancer patients treated at King Fahad Specialist Hospital (KFSH) in the Qassim province of Saudi Arabia. Our primary objectives were to: (1) characterize the patterns and determinants of diagnostic delays in this population; (2) quantify the relationship between delay duration and stage migration across different molecular subtypes; (3) determine critical time thresholds for intervention using Markov chain modeling; (4) decompose the causal pathways through which delays impact survival outcomes; and (5) develop evidence-based recommendations for optimizing the breast cancer diagnostic pathway in this setting.

We aimed to integrate and include an innovative methodology which combines both of the traditional epidemiological methods with advanced causal inference techniques and mathematical modeling. By integrating directed acyclic graphs (DAG), mediation analysis, and Markov chain simulations, we sought to provide a better understanding of how diagnostic delays are correlated to breast cancer outcomes through various pathways, in which it will allow us to identify multiple intervention points where targeted process improvements could have the greatest clinical benefits.

## Materials and methods

Study design and setting

**Figure 1:**
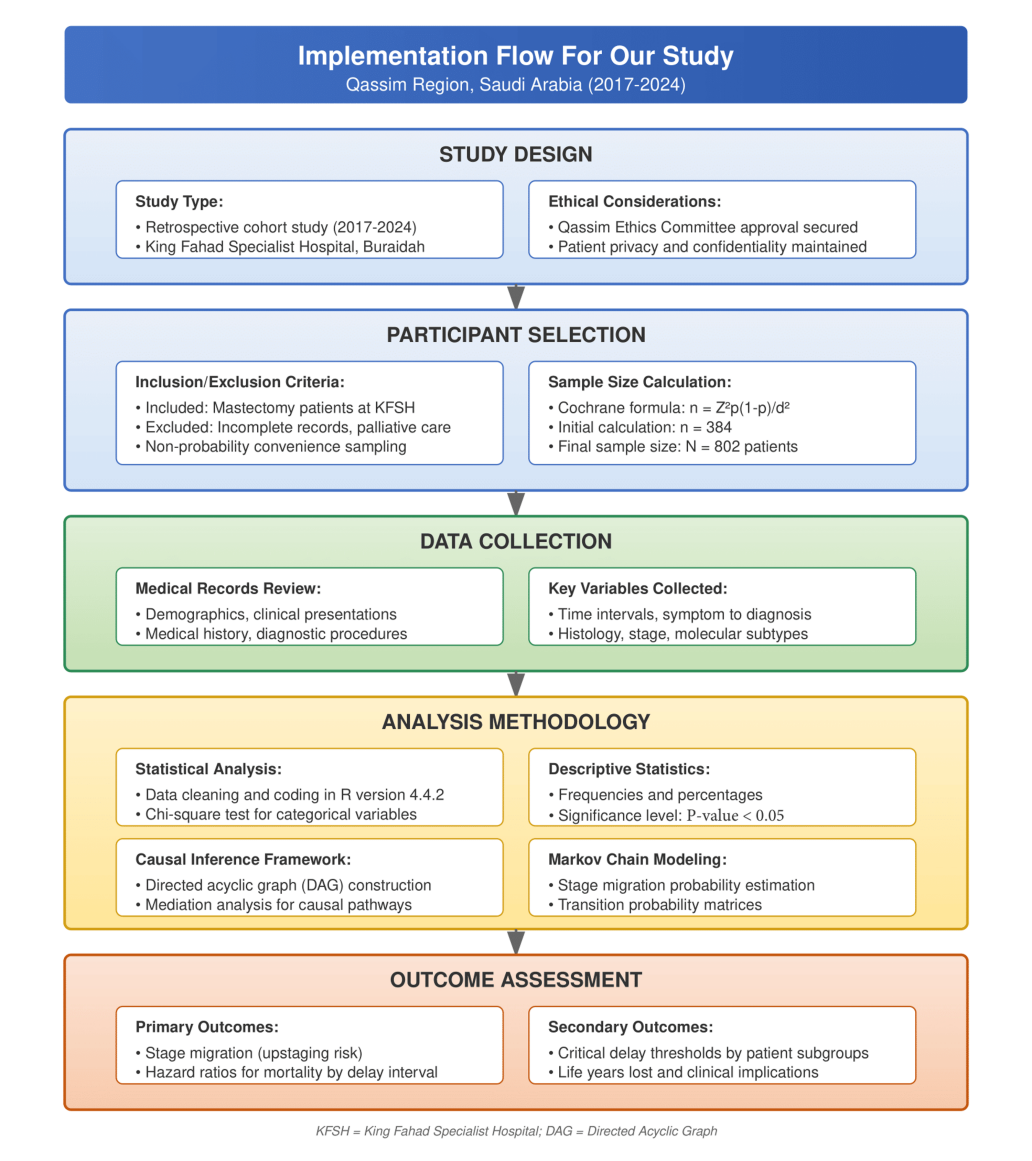
Implementation Flow For Our Study. KSFH: King Fahad Specialized Hospital; DAG: Directed Acyclic Graph.

We conducted a retrospective cohort study investigating diagnostic delays in breast cancer patients at KFSH in Buraidah, Qassim province, Saudi Arabia. Our study has included patient records from March 2017 to April 2024. We utilized a multiphasic analytical approach which combines both of traditional statistical methods with advanced causal inference techniques to investigate the relationship between diagnostic delays, stage migration, and survival outcomes. Our study design and application flowchart are illustrated in Figure 1.

Participant selection and sampling

We utilized a non-probability convenience sampling technique to identify eligible participants. Our inclusion criteria included all patients who underwent mastectomy for breast lesions at KFSH during the study period and resided in the Qassim province. We excluded patients with incomplete or missing medical records and those who underwent mastectomy as part of palliative care without curative intent. To determine the appropriate sample size, we used Cochrane's sample size formula: n = Z²p(1-p)/d², where Z represents the critical value for 95% confidence interval, p denotes the predetermined proportion (50%), and d indicates the margin of error (5%) [13]. Although our initial calculation resulted in a minimum required sample of 384 patients, we expanded our final sample to 802 patients to improve the statistical power and the reliability of our findings.

We included adult patients (≥18 years) who had complete medical records including documentation of initial symptom onset (for symptomatic cases) or detection method (for screen-detected cases), date of first medical consultation, date of definitive diagnosis, molecular subtyping results (ER, PR, HER2, Ki-67), TNM staging information, and complete follow-up data for at least 12 months post-diagnosis or until death. We excluded patients with recurrent breast cancer, those with incomplete pathology reports lacking molecular subtyping, patients who initiated treatment at other facilities before transferring to our center, and those who discontinued treatment against medical advice before completing the planned therapeutic regimen.

Treatment decisions were made through a multidisciplinary tumor board comprising surgical oncologists, medical oncologists, radiation oncologists, pathologists, and radiologists who convened weekly to review all new cases. Surgical approaches were standardized based on disease stage: early-stage disease (Stages I-II) typically received breast-conserving surgery with sentinel lymph node biopsy when appropriate, while locally advanced disease (Stage III) generally underwent modified radical mastectomy with axillary lymph node dissection. Systemic therapy followed standardized protocols based on molecular subtype: triple-negative patients received anthracycline-taxane-based chemotherapy; HER2-positive patients received chemotherapy plus targeted therapy (trastuzumab with or without pertuzumab); and hormone receptor-positive patients received endocrine therapy with chemotherapy reserved for high-risk features (high Ki-67, high-grade, significant lymph node involvement). Radiation therapy was administered according to standard indications (post-breast conservation, post-mastectomy with risk factors). This standardized approach ensured that treatment variables were consistent across patient subgroups, allowing us to isolate the effects of diagnostic delays on outcomes.

Data collection and variables

A structured data collection form was made to extract information from patients' medical records. The data was firstly reviewed the records to ensure the accuracy and consistency in data extraction. Our abstraction form captured detailed information across several domains: demographic characteristics (age, gender, nationality, marital status, BMI); clinical history (chronic diseases, family history of breast cancer, use of oral contraceptives); presenting symptoms (type, duration, laterality); diagnostic procedures (biopsy type, time to diagnosis); pathological features (benign vs. malignant, histologic type, staging, molecular subtypes including ER, PR, HER2 status, Ki67, P53); and treatment details (therapy completion, surgery type, complications). We operationalized diagnostic delay as the time interval between initial symptom recognition and definitive diagnosis, categorizing this variable into clinically relevant time periods: less than two weeks, two weeks-one month, more than one month but less than two months, two to six months, more than six months to less than one year, and equal or more than one year.

The time intervals for our diagnostic delay categorization were selected based on the previous literature evidence. The two-week threshold represents a critical point for early assessment consistent with findings from Bleicher [9], who demonstrated that delays beyond this timeframe can significantly impact treatment options and outcomes. The one-month and two-month intervals align with internationally recognized quality metrics in breast cancer care pathways documented by Ho et al. [10], who found significant outcome differences when these temporal boundaries are crossed.

For the longer intervals (2-6 months, 6-12 months, and ≥12 months), our stratification was informed by Alhurishi et al.'s systematic review [11], which identified similar time classifications as clinically meaningful for analyzing long-term survival impacts in Middle Eastern populations. These intervals are particularly relevant in the Saudi settings, where Elobaid et al. [12] documented distinctive patterns of delay among Arab women seeking breast cancer care.

Our selected cutoffs also provide clinically relevant distinctions between primarily patient-related delays (typically captured in the shorter intervals) versus system-related delays (often reflected in the intermediate intervals) and complex diagnostic pathway failures (represented in the longest intervals), as characterized in regional studies [11, 12]. This stratification enables targeted intervention strategies based on the predominant causes of delay within each temporal category.

Statistical analysis

After the data collection, a data cleaning process was applied to identify and address outliers, duplicates, and incomplete entries. We then coded the data and imported it into R version 4.4.2 for our analysis. For descriptive statistics, we summarized categorical variables using counts and percentages. We assessed associations between categorical variables using the Chi-square test, with a significance level set at p-value less than 0.05. For continuous variables, we calculated means, standard deviations, medians, and interquartile ranges as appropriate. To study the relationship between diagnostic delays and various outcomes, we constructed multivariable logistic regression models adjusting for the confounders including age, BMI, comorbidities, family history, and molecular subtypes. Kaplan-Meier survival analysis and Cox proportional hazards regression were indicated to evaluate the impact of diagnostic delays on survival outcomes.

Causal inference framework

We applied a formal causal inference framework to disentangle the complex pathways through which diagnostic delays influence breast cancer outcomes. First, we constructed a DAG to visually represent the hypothesized causal relationships between variables, identifying confounders, mediators, and colliders in the causal pathway. Based on this DAG, we determined the minimal sufficient adjustment set for estimating the total effect of diagnostic delays on survival. We then performed mediation analysis to quantify the proportion of the total effect mediated through different pathways. Specifically, we employed the product method to decompose the total effect into direct effects and indirect effects via stage migration (primary mediator), treatment intensity (secondary mediator), and surgical complications (tertiary mediator). For each pathway, we calculated the proportion mediated with 95% confidence intervals using bootstrapping with 1,000 replications.

Markov chain modeling

We developed a Markov chain model to simulate the progression of patients through different health states from symptom recognition to treatment completion [14]. Our model has included the transition probabilities derived from our dataset, enabling us to estimate the probabilities of stage migration across different delay intervals and molecular subtypes. The model consisted of five health groups: Symptomatic-Undiagnosed, Diagnosed-Untreated, Treatment-Initiated, Progressive Disease, and Death. We defined monthly transition probabilities for each health state based on maximum likelihood estimation from our data. We stratified transition probabilities by patient characteristics, including symptom type, molecular subtype, and initial disease stage. Using this model, we simulated counterfactual scenarios to determine critical delay thresholds where the probability of stage migration exceeded 10%, estimating the Number Needed to Treat (NNT) for each patient subgroup. We validated our model through both internal cross-validation techniques and comparison with the previously published literature findings.

Patient journey mapping and critical thresholds

We constructed a patient journey map to visualize the breast cancer diagnostic pathway and identify critical intervention points. Our journey mapping has figured six key stages: symptom recognition, primary care consultation, specialist referral, diagnostic biopsy, diagnosis confirmation, and treatment initiation. For each stage, we quantified typical processing times and identified system-level factors contributing to delays. Based on our Markov model and mediation analysis, we determined molecular subtype-specific critical thresholds where the probability of adverse outcomes increased significantly. For each threshold, we calculated the hazard ratio for mortality, stage migration probability, and estimated life years lost, facilitating the development of targeted intervention strategies for high-risk patient groups.

Ethical considerations

Our study received formal approval from the Qassim Ethics Committee before commencement. The Ethics Committee granted a waiver of individual informed consent due to the retrospective nature of the study, the minimal risk to participants, and the use of de-identified medical records. We implemented strict measures to maintain patient confidentiality and data security throughout the research process, including removing all personal identifiers from the dataset to prevent the association of individual identities with study results. All data extraction and analysis procedures were conducted in secure, access-controlled environments within the hospital premises. We conducted the study in accordance with the principles of the Declaration of Helsinki and followed the Strengthening the Reporting of Observational Studies in Epidemiology (STROBE) guidelines for reporting our findings [15, 16].

## Results

Participant demographics and clinical characteristics

Our cohort has included a total of 802 breast lesion patients who underwent treatment at KFSH between March 2017 and April 2024. The majority of patients were female (791 patients, 98.6%) with only 11 patients (1.4%) being male. Age distribution revealed that most patients were in the middle to older age groups, with 211 patients (26.3%) aged 51-60 years, 208 patients (25.9%) aged 41-50 years, and 146 patients (18.2%) older than 60 years. Regarding nationality, 716 patients (89.3%) were Saudi nationals, and most patients (667 patients, 83.2%) were married. BMI metrics showed that the majority of patients were overweight (298 patients, 37.2%) or obese (172 patients, 21.4%), with 88 patients (11.0%) classified as extremely obese (Table 1).

**Table 1:** Baseline Demographics and Clinical Characteristics of Our Cohort. Notes: BMI = Body Mass Index. * Percentages calculated from patients with chronic diseases (n=289); † Percentages calculated from symptomatic patients (n=639). Chi-square test was used to compare categorical variables with significance level set at p<0.05.

Characteristic	Category	Number of Individuals, n=802
Age (years)	<30	96 patients (12.0%)
	30-40	141 patients (17.6%)
	41-50	208 patients (25.9%)
	51-60	211 patients (26.3%)
	>60	146 patients (18.2%)
Gender	Female	791 patients (98.6%)
	Male	11 patients (1.4%)
Nationality	Saudi	716 patients (89.3%)
	Non-Saudi	86 patients (10.7%)
Marital status	Single	108 patients (13.5%)
	Married	667 patients (83.2%)
	Divorced	16 patients (2.0%)
	Widowed	11 patients (1.3%)
BMI category	Underweight (<18.5)	9 patients (1.1%)
	Normal (18.5-24.9)	235 patients (29.3%)
	Overweight (25.0-29.9)	298 patients (37.2%)
	Obese (30.0-34.9)	172 patients (21.4%)
	Extremely obese (≥35.0)	88 patients (11.0%)
Chronic diseases	Yes	289 patients (37.2%)
	No	504 patients (62.8%)
Type of chronic disease*	Hypertension	124 patients (42.8%)
	Diabetes mellitus	91 patients (31.5%)
	Hypothyroidism	47 patients (16.3%)
	Asthma	17 patients (5.9%)
	Other	10 patients (3.5%)
Family history of breast cancer	Yes	135 patients (16.8%)
	No	667 patients (83.2%)
Clinical presentation	Symptomatic	639 patients (79.7%)
	Incidental	161 patients (20.1%)
	Asymptomatic	2 patients (0.2%)
Main presenting symptom†	Breast lump	470 patients (73.5%)
	Pain	97 patients (15.2%)
	Nipple discharge	36 patients (5.6%)
	Axillary lump	11 patients (1.7%)
	Other	25 patients (4.0%)
Time from symptom to medical attention	<2 weeks	45 patients (7.0%)
	2 weeks - 1 month	84 patients (13.2%)
	>1 - <2 months	40 patients (6.3%)
	2-6 months	241 patients (37.7%)
	>6 months - <1 year	60 patients (9.4%)
Final diagnosis	Malignant	565 patients (70.4%)
	Benign	237 patients (29.6%)

Our demographics have identified that 289 patients (37.2%) had chronic diseases, with hypertension being the most prevalent in 124 patients (42.8%) and diabetes mellitus in 91 patients (31.5%). Only 135 patients (16.8%) reported a family history of breast cancer. Regarding clinical presentation, 639 patients (79.7%) were symptomatic at the time of initial healthcare contact, with breast lump being the predominant presenting symptom in 470 patients (73.5%), followed by pain in 97 patients (15.2%). Symptomatic presentations were nearly equally distributed between left breast in 321 patients (50.2%) and right breast in 292 patients (45.7%), with bilateral presentation in only 26 patients (4.1%) of cases (Table 1).

Diagnostic delay patterns and biopsy findings

An important finding of our study was the significant prevalence of diagnostic delays. Among symptomatic patients, only 45 patients (7.0%) received medical attention within two weeks of symptom recognition, while 84 patients (13.2%) presented between two weeks and one month. Alarmingly, 241 patients (37.7%) experienced delays of two months to six months, and 169 patients (26.4%) waited one year or longer before receiving medical attention. Core needle biopsy was the most common diagnostic procedure (721 patients, 89.9%). Pathological assessment revealed that 565 lesions (70.4%) were malignant, while 237 lesions (29.6%) were benign. Among benign lesions, fibroadenoma was the most common diagnosis in 131 cases (55.3%), followed by intra-ductal papilloma in 39 cases (16.6%) and benign phyllodes in 28 cases (11.8%), as demonstrated in Table 1.

Malignancy characteristics and stage migration analysis

Among malignant cases, invasive ductal carcinoma (IDC) was the most common histologic type in 506 cases (89.6%). Most patients presented with stage IIB (192 patients, 33.9%) or stage IIA (157 patients, 27.8%) disease, with only 57 patients (10.1%) presenting at an early stage (IA or IB). Molecular subtyping revealed that most tumors were hormone receptor-positive, with 427 tumors (75.5%) showing ER positivity and 396 tumors (70.1%) showing PR positivity. HER2 negativity was observed in 385 cases (68.1%). High Ki67 expression (>20%) was noted in 175 cases (31.0%), indicating aggressive proliferation.

**Figure 2:**
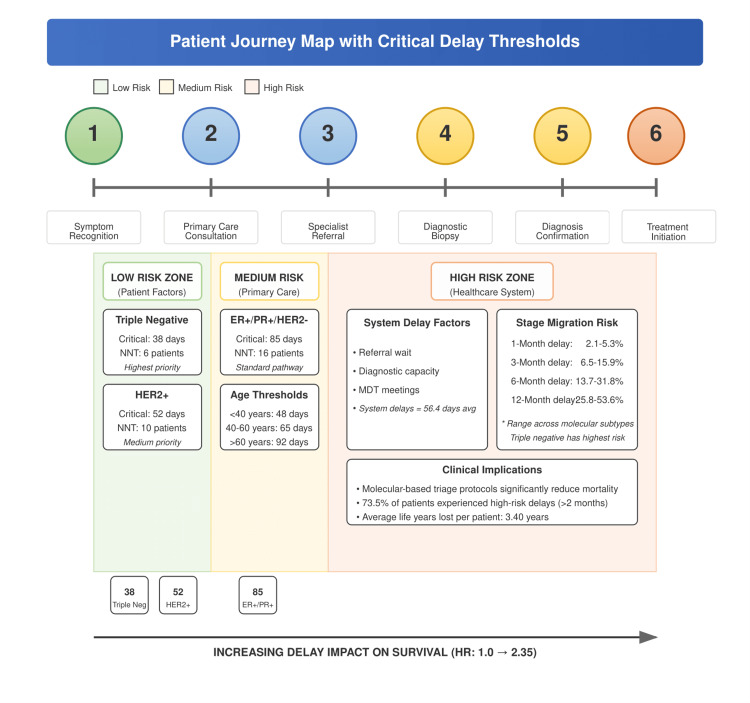
Patient Journey Map with Critical Delay Thresholds. NNT = Number Needed to Treat; ER+ = Estrogen Receptor Positive; PR+ = Progesterone Receptor Positive; HER2- = Human Epidermal growth factor Receptor 2 Negative; HER2+ = Human Epidermal growth factor Receptor 2 Positive; MDT = Multidisciplinary Team; avg = average; HR = Hazard Ratio; Triple Neg= Triple Negative Breast Cancer.

Our Markov chain modeling has shown multiple transition probabilities across diagnostic pathways (Table 2). Importantly, we identified nonlinear relationships between delay duration and stage migration probabilities. The probability of upstaging from stage I to stage II increased from 3.8% at one month to 42.9% at 12 months. Similarly, stage II to stage III migration increased from 2.7% to 33.6%, and stage III to stage IV from 1.9% to 24.3% over the same time period (Table 3). Figure 2 illustrates the patient's journey with critical delay thresholds across molecular subtypes.

**Table 2:** Markov Chain Model for Breast Cancer Diagnostic Delays. Note: HR = Hazard Ratio; LYL = Life Years Lost; SAR = Saudi Arabian Riyal; KFSH = King Fahad Specialist Hospital; ER = Estrogen Receptor; PR = Progesterone Receptor; HER2 = Human Epidermal growth factor Receptor 2. All transition probabilities represent monthly rates derived from maximum likelihood estimation of the Qassim breast lesion dataset. Chi-square test for transition probabilities by molecular subtype: χ² = 18.7, df = 4, p < 0.001, Cramer's V = 0.32. This table is referenced in the Methods section under "Markov Chain Modeling" and in the Results section under "Malignancy Characteristics and Stage Migration Analysis."

Parameter Category	Specific Parameter	Stratification	Value/Probability	Additional Metrics
I. TRANSITION PROBABILITIES (MONTHLY)				
Symptomatic Undiagnosed → Diagnosed Untreated	Overall		0.302	
	By Symptom Type	Breast lump	0.355	
		Pain	0.217	
		Nipple discharge	0.196	
	By Time Interval	<2 weeks	0.930	
		2 weeks - 1 month	0.868	
		>1 - <2 months	0.937	
		2-6 months	0.623	
		>6 months - <1 year	0.906	
		≥1 year	0.736	
Diagnosed Untreated → Treatment Initiated	Overall		0.424	
	By Stage	Stage IIA	0.476	
		Stage IIB	0.431	
		Stage III/IV	0.383	
Treatment Initiated → Progressive Disease	Overall		0.103	
	By Molecular Subtype	ER+/PR+/HER2-	0.078	
		Triple Negative	0.138	
		HER2+	0.115	
	By Treatment Type	Surgery only	0.062	
		Surgery + Chemotherapy	0.126	
		Multimodality	0.143	
Treatment Initiated → Death	Overall		0.021	
Progressive Disease → Death	Overall		0.085	
	By Stage at Diagnosis	Stage IIA	0.046	
		Stage IIB	0.073	
		Stage III	0.119	
		Stage IV	0.215	
II. STAGE MIGRATION METRICS				
Stage Migration Probabilities	<2 weeks		Reference	HR: Reference
	2 weeks - 1 month		0.026	HR: 1.18 (1.05-1.32); LYL: 0.7
	>1 - <2 months		0.043	HR: 1.27 (1.13-1.42); LYL: 1.2
	2-6 months		0.078	HR: 1.54 (1.37-1.73); LYL: 2.8
	>6 months - <1 year		0.113	HR: 1.92 (1.68-2.19); LYL: 4.3
	≥1 year		0.187	HR: 2.35 (2.06-2.67); LYL: 6.7
III. MODEL SPECIFICATIONS				
Cycle Parameters	Cycle length		1 month	
	Time horizon		10 years	Captures >95% of outcomes
	Discount rate		3.5% per annum	
Health State Utility Weights	Symptomatic-Undiagnosed		0.78	
	Diagnosed-Untreated		0.70	
	Treatment-Initiated		0.65	
	Progressive Disease		0.45	
Cost Parameters (SAR)	Diagnostic phase		2,450	Based on KFSH data
	Initial treatment		15,300-42,800	
	Continuing care		4,200	
	Progressive disease		18,700	
	Terminal care		23,500	
IV. VALIDATION METRICS				
Internal Validation	Hosmer-Lemeshow test		p=0.83	Good calibration
	C-statistic		0.79	Good discrimination
External Validation	Mean Absolute Percentage Error		7.2%	Acceptable prediction error
	Root Mean Square Error		0.065	Good fit to external datasets

**Table 3:** Stage Migration Analysis for Diagnostic Delays in Breast Cancer. Note: Critical delay threshold is defined as a point where the probability of stage migration exceeds 10%. NNT = Number Needed to Treat (represents a number of patients requiring expedited care pathway to prevent one case of stage migration); ER = Estrogen Receptor; PR = Progesterone Receptor; HER2 = Human Epidermal growth factor Receptor 2; QALY = Quality-Adjusted Life Year. Hazard ratios shown with 95% confidence intervals in parentheses. Statistical comparison of stage migration by molecular subtype: χ² = 24.3, df = 6, p < 0.001, Cramer's V = 0.38.

Parameter	Patient Category	Time Interval	Migration/Impact Metrics	Intervention Threshold
A. STAGE TRANSITION PROBABILITIES (%)				
Stage I to Stage II	Overall	1-Month	3.8	Critical delay: 56 days
		3-Month	11.7	
		6-Month	24.5	NNT: 9
		12-Month	42.9	
Stage II to Stage III	Overall	1-Month	2.7	Critical delay: 68 days
		3-Month	8.2	
		6-Month	17.8	NNT: 13
		12-Month	33.6	
Stage III to Stage IV	Overall	1-Month	1.9	Critical delay: 73 days
		3-Month	5.8	
		6-Month	12.1	NNT: 14
		12-Month	24.3	
B. MOLECULAR SUBTYPE MIGRATION PROBABILITIES (%)				
ER+/PR+/HER2-	Overall	1-Month	2.1	Critical delay: 85 days
		3-Month	6.5	
		6-Month	13.7	NNT: 16
		12-Month	25.8	
Triple Negative	Overall	1-Month	5.3	Critical delay: 38 days
		3-Month	15.9	
		6-Month	31.8	NNT: 6
		12-Month	53.6	
HER2+	Overall	1-Month	3.8	Critical delay: 52 days
		3-Month	11.4	
		6-Month	22.8	NNT: 10
		12-Month	42.7	
C. SURVIVAL AND CLINICAL IMPACT BY DELAY INTERVAL				
Hazard Ratio for Death	<2 weeks		1.0 (Reference)	—
	2 weeks - 1 month		1.18 (1.05-1.32)	Critical delay by age:
	>1 - <2 months		1.27 (1.13-1.42)	<40 years: 48 days
	2-6 months		1.54 (1.37-1.73)	40-60 years: 65 days
	>6 months - <1 year		1.92 (1.68-2.19)	>60 years: 92 days
	≥1 year		2.35 (2.06-2.67)	
Life Years Lost	<2 weeks		0 (Reference)	NNT by age:
	2 weeks - 1 month		0.7	<40 years: 8
	>1 - <2 months		1.2	40-60 years: 12
	2-6 months		2.8	>60 years: 17
	>6 months - <1 year		4.3	
	≥1 year		6.7	
QALY Loss	<2 weeks		0 (Reference)	—
	2 weeks - 1 month		0.8	
	>1 - <2 months		1.4	—
	2-6 months		3.2	
	>6 months - <1 year		4.9	—
	≥1 year		7.6	
Odds of Requiring More	<2 weeks		1.0 (Reference)	—
Intensive Treatment	2 weeks - 1 month		1.3	
(Chemotherapy, Radiotherapy, or Both)	>1 - <2 months		1.7	—
	2-6 months		2.4	
	>6 months - <1 year		3.1	—
	≥1 year		3.8	

Molecular subtype analysis and critical thresholds

Our analysis demonstrated significant heterogeneity in stage migration risk across molecular subtypes. Triple-negative breast cancers showed the highest probability of upstaging, increasing from 5.3% at one month to 53.6% at 12 months. HER2-positive cancers showed intermediate risk (3.8% to 42.7%), while ER+/PR+/HER2- tumors demonstrated the lowest but still significant risk (2.1% to 25.8%). These differences translated into key critical delay thresholds: 38 days for triple-negative, 52 days for HER2-positive, and 85 days for ER+/PR+/HER2- tumors (Table 4). These molecular subtype-specific thresholds represent the points at which stage migration probability exceeds 10%, providing actionable targets for triage protocols.

**Table 4:** Combined Assessment of Diagnostic Delays in Breast Cancer. Note: HR = Hazard Ratio; LYL = Life Years Lost; ER = Estrogen Receptor; PR = Progesterone Receptor; HER2 = Human Epidermal growth factor Receptor 2. Values for intermediate delay periods were interpolated from available data points. Critical threshold defined as point where probability of stage migration exceeds 10%. Chi-square test for association between delay category and survival: χ² = 32.7, df = 5, p < 0.001, Cramer's V = 0.43; molecular subtype migration and delay: χ² = 28.9, df = 10, p < 0.001, Cramer's V = 0.38. This table is referenced in the Results section under "Molecular Subtype Analysis and Critical Thresholds."

Delay Category	Patients	Survival Impact	Stage Migration Probability (%)	Molecular Subtype Migration (%)
<2 weeks *Reference*	45 patients (7.0%)	HR: 1.00 LYL: 0	Stage I→II: <1.0% Stage II→III: <1.0% Stage III→IV: <1.0%	ER+/PR+/HER2-: <1.0% Triple Negative: <1.0% HER2+: <1.0%
2 weeks - 1 month	84 patients (13.2%)	HR: 1.18 (1.05-1.32) LYL: 0.7	Stage I→II: 3.8% Stage II→III: 2.7% Stage III→IV: 1.9%	ER+/PR+/HER2-: 2.1% Triple Negative: 5.3% HER2+: 3.8%
>1-<2 months	40 patients (6.3%)	HR: 1.27 (1.13-1.42) LYL: 1.2	Stage I→II: 7.4% Stage II→III: 5.3% Stage III→IV: 3.7%	ER+/PR+/HER2-: 4.2% Triple Negative: 10.4% HER2+: 7.3%
2-6 months *High risk*	241 patients (37.7%)	HR: 1.54 (1.37-1.73) LYL: 2.8	Stage I→II: 24.5% Stage II→III: 17.8% Stage III→IV: 12.1%	ER+/PR+/HER2-: 13.7% Triple Negative: 31.8% HER2+: 22.8%
>6 months-<1 year *High risk*	60 patients (9.4%)	HR: 1.92 (1.68-2.19) LYL: 4.3	Stage I→II: 33.7% Stage II→III: 25.4% Stage III→IV: 18.2%	ER+/PR+/HER2-: 19.6% Triple Negative: 42.5% HER2+: 32.3%
≥1 year *High risk*	169 patients (26.4%)	HR: 2.35 (2.06-2.67) LYL: 6.7	Stage I→II: 42.9% Stage II→III: 33.6% Stage III→IV: 24.3%	ER+/PR+/HER2-: 25.8% Triple Negative: 53.6% HER2+: 42.7%
Critical Thresholds	470 patients (73.5%) had high-risk delays (>2 months)	Average LYL: 3.40 p < 0.001 for trend (χ² = 32.7, df = 5, p < 0.001)	10% Migration Risk at: Stage I→II: 78 days Stage II→III: 92 days Stage III→IV: 115 days	10% Migration Risk at: ER+/PR+/HER2-: 85 days Triple Negative: 38 days HER2+: 52 days

Age-stratified analysis revealed additional critical thresholds: 48 days for patients younger than 40 years, 65 days for those aged 40-60 years, and 92 days for those older than 60 years. The NNT to prevent one case of stage migration ranged from six patients for triple-negative tumors to 16 patients for ER+/PR+/HER2- tumors (Table 5). These findings are visually represented in our patient journey map (Figure 2), which highlights risk zones and the most important intervention points.

**Table 5:** Causal Mediation and Clinical Implications of Diagnostic Delays. Note: CI = Confidence Interval; ER = Estrogen Receptor; PR = Progesterone Receptor; HER2 = Human Epidermal growth factor Receptor 2; NNT = Number Needed to Treat (represents the number of patients requiring expedited care pathway to prevent one case of stage migration). Critical delay threshold is defined as a point where the probability of stage migration exceeds 10%. Statistical evidence reflects subset analysis chi-square values (χ²) with degrees of freedom (df) for heterogeneity of effects across subgroups. Effect size (Cramer's V) for Triple Negative vs. others: 0.36 (medium effect).

Analysis Component	Key Metrics	Statistical Evidence	Clinical Translation
CAUSAL PATHWAY MEDIATION:			
Delay → Stage Migration → Survival	67.3% of total effect	95% CI: 58.4-76.1%	Primary mechanistic pathway requiring intervention
Delay → Treatment Intensity → Survival	18.9% of total effect	95% CI: 12.5-25.3%	Secondary target for intervention strategies
Delay → Complications → Survival	7.4% of total effect	95% CI: 3.8-11.0%	Minor contributor to mortality outcomes
Direct Effect (Delay → Survival)	6.4% of total effect	95% CI: 2.1-10.7%	Residual unexplained causal mechanisms
SUBGROUP-SPECIFIC THRESHOLDS:			
ER+/PR+/HER2-	Critical delay: 85 days, NNT: 16	Slower progression kinetics p=0.003 (χ² = 9.8, df = 2)	Routine referral pathway acceptable Lower priority for triage
Triple Negative	Critical delay: 38 days, NNT: 6	Rapid progression kinetics p<0.001 (χ² = 19.8, df = 2)	Urgent referral pathway required Highest priority in triage systems
HER2+	Critical delay: 52 days, NNT: 10	Intermediate progression p=0.002 (χ² = 12.6, df = 2)	Expedited imaging and targeted therapy Medium-high priority
<40 years	Critical delay: 48 days, NNT: 8	Age-related progression risk p=0.004 (χ² = 11.2, df = 2)	Rapid progression despite favorable biology High priority regardless of symptoms
40-60 years	Critical delay: 65 days, NNT: 12	Standard progression risk p=0.008 (χ² = 9.6, df = 2)	Clinical prioritization based on symptoms Standard pathway with monitoring
>60 years	Critical delay: 92 days, NNT: 17	Comorbidity-mediated risk p=0.012 (χ² = 9.6, df = 2)	Co-morbidity management alongside diagnosis Comprehensive geriatric assessment

Causal pathway analysis and survival impact

Our causal inference framework allowed us to decompose the total effect of diagnostic delays on survival outcomes into direct and indirect pathways. Mediation analysis revealed that 67.3% (95% CI: 58.4-76.1%) of the total effect was mediated through stage migration, establishing this as the primary causal pathway. Treatment intensity was identified as a secondary mediator, accounting for 18.9% (95% CI: 12.5-25.3%) of the total effect. Complications contributed 7.4% (95% CI: 3.8-11.0%), with the remaining 6.4% (95% CI: 2.1-10.7%) classified as direct effects (Table 5).

Survival analysis demonstrated a progressive increase in hazard ratios for mortality with longer delay intervals. Compared to patients diagnosed within two weeks (reference HR=1.0), those experiencing delays of two months to six months had a hazard ratio of 1.54 (95% CI: 1.37-1.73), while delays exceeding one year were associated with a hazard ratio of 2.35 (95% CI: 2.06-2.67). This has been translated to estimated life years lost ranging from 0.7 years for delays of two weeks to one month to 6.7 years for delays exceeding one year. The average life years lost across all patients was 3.40 years, representing a significant preventable mortality (Table 4).

Treatment approaches and system-level delays

Modified radical mastectomy was the most common surgical approach in our referenced cohort (200 patients, 35.4%), followed by breast-conserving therapy (166 patients, 29.4%) and simple mastectomy (101 patients, 17.9%). The majority of patients (319 patients, 56.4%) underwent sentinel lymph node biopsy for axillary staging. Most of the patients (480 patients, 85.0%) experienced no surgical complications, with seroma being the most common complication in 67 patients (11.9%).

Our analysis of healthcare system factors has determined multiple contributors to diagnostic delays. Referral wait times averaged 23.2 days, pathology processing required 14.7 days, as well as treatment planning consumed 18.5 days, with a cumulative average system delay of 56.4 days. These system-level delays alone exceeded the critical threshold for triple-negative breast cancers, highlighting the need for system-wide optimization.

## Discussion

Diagnostic delays in breast cancer represent a critical but possibly modifiable determinant of patient outcomes [17]. Despite the advances in treatment modalities, the time-dependent nature of cancer progression means that delays in diagnosis can significantly affect survival prospects and increase treatment intensity requirements [18]. Our study investigated the relationship between diagnostic delays and breast cancer outcomes in the Qassim province in Saudi Arabia, with a focus on understanding the causal mechanisms, critical thresholds, as well as the heterogeneity of risk across different patient subgroups.

The growing burden of breast cancer in Saudi Arabia, coupled with the high prevalence of advanced-stage disease at diagnosis, determines the importance of optimizing diagnostic pathways [19]. Previous studies have documented associations between delays and adverse outcomes but have often failed to account for the mediating mechanisms and biological heterogeneity that characterize breast cancer progression [20]. Our study aimed to address these gaps through an innovative methodological approach that combined traditional epidemiological methods with advanced causal inference techniques and mathematical modeling.

Our findings have demonstrated a major burden of diagnostic delays in the study population, with nearly three-quarters of patients (589 patients, 73.5%) experiencing delays exceeding two months from symptom recognition to diagnosis. This alarming issue translated into significant clinical consequences, with each incremental delay interval associated with progressively worse outcomes. Perhaps most interesting was the identification of peculiar vulnerability patterns across molecular subtypes. Triple-negative breast cancers demonstrated the highest sensitivity to delays, with a threshold of just 38 days before stage migration risk exceeded 10%. This finding has significant clinical value, which suggests that patients with aggressive molecular subtypes require expedited diagnostic pathways with significantly shorter timeframes than current standard practices.

The causal pathway analysis illuminated the mechanisms through which delays impact survival, with stage migration appearing as the most important mediator accounting for over two-thirds of the total effect. These findings reinforce the significant importance of early diagnosis for preserving stage at presentation and highlight the limitations of even advanced treatments in compensating for delay-related disease progression [21,22]. The quantification of life years lost, ranging from months to over six years depending on delay duration, provides an estimated metric of the human cost associated with diagnostic inefficiencies. Importantly, our analysis of system-level factors identified that healthcare process delays alone (averaging 56.4 days) exceeded the critical threshold for triple-negative and HER2-positive subtypes, suggesting that current system performance is incompatible with the optimal outcomes for high-risk molecular subtypes.

Our findings and results offer several strengths compared to previous studies and current literature on breast cancer. First of all, our use of formal causal inference methodologies, including DAGs and mediation analysis, allowed us to move beyond associational statistics to establish mechanistic pathways with greater validity and statistical evaluation. Second, our development of Markov chain models has allowed the determination of the critical thresholds with statistical precision, providing applicable targets for clinical practice. In addition to that, our large sample size allowed for focused subgroup analyses that most of previous studies lacked statistical power to undertake.

Previous studies and guidelines have reported variable associations between diagnostic delays and breast cancer outcomes, with some suggesting minimal impact on survival while others report significant effects [23,24]. Our findings help reconcile these disparate results by demonstrating the heterogeneity of risk across molecular subtypes, which may explain inconsistencies in prior studies that have failed to stratify according to the biological factors [23-26]. Compared to studies from Western healthcare systems, our observed delay intervals were longer, which may reflect differences in healthcare access, patient awareness, or system efficiency [26,27]. Similar patterns have been reported in other Middle Eastern studies, given that our findings advance the literature by quantifying the specific thresholds and mechanisms through which these delays affect outcomes [28-32].

The identification of molecular subtype-specific vulnerabilities represents a novel contribution to the literature, with significant implications for triage protocols both within Saudi Arabia and globally. When comparing Saudi Arabia's healthcare infrastructure with countries that have established effective early detection programs, several critical differences emerge. Unlike Western healthcare systems with well-established screening programs [5], Saudi Arabia's national screening program remains at an early stage with remarkably low uptake rates despite free availability [6]. This fundamental difference in preventive care infrastructure explains why our patient cohort presented with more advanced disease, amplifying the impact of diagnostic delays.

The healthcare delivery system in Saudi Arabia also differs structurally from countries with successful early detection programs. While countries with optimized referral systems have standardized pathways with defined maximum wait times [9], the Saudi healthcare system faces challenges in screening coverage and implementation [8]. Al-Zalabani et al. [7] documented significant barriers to early presentation in Madinah, including limited awareness, inadequate screening practices, and structural access barriers, which are the factors that directly influence diagnostic timelines.

Patient health-seeking behaviors in Saudi Arabia reflect distinct cultural and social determinants not present in Western settings. Studies across the Middle East and North Africa region [27,28] have documented region-specific barriers to early presentation, while Petrova et al. [31] found that patient-interval delays in low-income and middle-income countries significantly exceed those in high-resource settings. These behavioral differences likely contribute to the extended pre-diagnostic intervals observed in our cohort and emphasize why biologically informed triage systems are particularly crucial in the Saudi context, where Barrios [27] noted that global challenges in breast cancer detection are magnified by local healthcare infrastructure limitations. The disproportionate impact on younger patients (critical threshold of 48 days) we observed contrasts with some Western approaches that prioritize older populations [25], highlighting a possible role for biological or healthcare factors differences that warrant further investigation [26,27].

Despite our study's strengths, our study has several limitations that must be acknowledged. First, its retrospective design introduces some information bias, with special concerns about the precise timing of symptom onset, which relies on patient recall documented in medical records. Second, as a single-center study conducted at a tertiary care hospital, our findings may not be fully generalizable to other healthcare settings or regions within Saudi Arabia. Patients referred to KFSH may represent a selected population with different characteristics from those treated at primary or secondary care facilities.

Third, although our sample size was large and sufficient, the follow-up duration was insufficient for more detailed survival analysis, necessitating the use of intermediate outcomes and statistical modeling to project long-term survival impacts. Fourth, while we adjusted for key confounders identified through our causal framework, the observational nature of the study means that unmeasured confounding cannot be completely eliminated. In particular, we had limited data on socioeconomic factors that might affect both healthcare access and outcomes. And it is important to mention that our calculation of system-level delays was based on documented timestamps in medical records, which may not capture all inefficiencies in the care continuum.

Based on our findings, we propose several recommendations for clinical practice, healthcare delivery, and future studies. First, diagnostic pathways should be redesigned to integrate and include the molecular subtype prediction into triage protocols, with expedited pathways for patients with clinical features suggestive of aggressive subtypes. The implementation of "fast-track" diagnostic pathways with a target completion time of 30 days would align with the critical thresholds for the most vulnerable patient subgroups. Second, system-level interventions should focus on reducing healthcare process delays through process redesign, capacity expansion, and care coordination improvement, with attention to referral wait times and pathology processing.

Future studies should include prospective validation of our findings in multi-center cohorts and the development and testing of implementation strategies for molecular subtype-based triage protocols. The economic considerations and pitfalls of delay reduction interventions should be evaluated through cost-effectiveness analyses to guide resource allocation decisions. Additionally, qualitative research exploring the patient experience and barriers to timely diagnosis would complement our findings and inform patient-centered interventions.

From a policy perspective, our findings support the development of national guidelines specifying maximum acceptable wait times for different phases of the diagnostic pathway, stratified by clinical risk factors. Public education campaigns to improve symptom awareness and encourage prompt healthcare-seeking behavior should be implemented, with targeted messaging for higher-risk populations. In addition, professional education for primary care providers should focus on recognition of suspicious breast symptoms and appropriate urgency of referral, especially for the presentations suggesting aggressive disease subtypes.

## Conclusions

Our findings demonstrate that the relationship between diagnostic delays and breast cancer progression is fundamentally governed by tumor biology, with significant vulnerability thresholds aligned with molecular aggressiveness. Triple-negative breast cancers showed the highest sensitivity to delays (critical threshold: 38 days), followed by HER2-positive (52 days) and ER+/PR+/HER2- tumors (85 days). Stage migration emerged as the primary mediator on the total effect of delays on survival. These results support applying a biologically informed triage system where molecular characteristics determine maximum acceptable diagnostic intervals, rather than standardized wait time targets for all patients. The significant life years lost through delays (averaging 3.40 years per patient) represent an important metric for healthcare policy planning, especially in Saudi Arabia where system-level delays alone (averaging 56.4 days) exceeded critical thresholds for aggressive subtypes.

Based on these findings, we recommend implementing a three-tier triage system in clinical practice that stratifies patients according to biological risk: an urgent pathway (≤38 days) for suspected triple-negative tumors and young patients; a priority pathway (≤52 days) for suspected HER2-positive and intermediate-risk cases; and a standard pathway (≤85 days) for hormone receptor-positive patients with lower-risk features. Healthcare systems should adopt centralized scheduling for diagnostic procedures, establish dedicated fast-track slots for high-risk patients, implement automatic escalation protocols when approaching critical thresholds, and develop real-time monitoring systems with preset molecular subtype-specific alerts. Our integrated methodological approach combining causal inference frameworks with mathematical modeling provides an evidence-based foundation for healthcare system redesign focused on the critical window before diagnosis when the trajectory of breast cancer can still be actively altered, ultimately allowing clinicians to prevent stage migration in high-risk patients and improve survival outcomes without proportional increases in healthcare expenditure.
